# The Antimelanoma Biological Assessment of Triterpenic Acid Functionalized Gold Nanoparticles

**DOI:** 10.3390/molecules28010421

**Published:** 2023-01-03

**Authors:** Marius Mioc, Alexandra Mioc, Roxana Racoviceanu, Roxana Ghiulai, Alexandra Prodea, Andreea Milan, Lucian Barbu Tudoran, Camelia Oprean, Viviana Ivan, Codruța Șoica

**Affiliations:** 1 Department of Pharmaceutical Chemistry, Faculty of Pharmacy, “Victor Babes” University of Medicine and Pharmacy, Eftimie Murgu Square No. 2, 300041 Timişoara, Romania; 2 Research Centre for Pharmaco-Toxicological Evaluation, “Victor Babes” University of Medicine and Pharmacy, Eftimie Murgu Square No. 2, 300041 Timisoara, Romania; 3 Department of Anatomy, Physiology, Pathophysiology, Faculty of Pharmacy, “Victor Babes” University of Medicine and Pharmacy, Eftimie Murgu Square No. 2, 300041 Timisoara, Romania; 4 Department of Pharmacology-Pharmacotherapy, “Victor Babes” University of Medicine and Pharmacy, Eftimie Murgu Square No. 2, 300041 Timisoara, Romania; 5 Electron Microscopy Laboratory “Prof. C. Craciun”, Faculty of Biology & Geology, “Babes-Bolyai” University, 5-7 Clinicilor Street, 400006 Cluj-Napoca, Romania; 6 Electron Microscopy Integrated Laboratory, National Institute for R & D of Isotopic and Molecular Technologies, 67-103 Donat Street, 400293 Cluj-Napoca, Romania; 7 Department of Chemistry and Toxicology, OncoGen Centre, County Hospital ‘Pius Branzeu’, Blvd. Liviu Rebreanu 156, 300736 Timisoara, Romania; 8 Department of Drug Analysis, Food and Environmental Chemistry, Legislation, Management and Pharmaceutical Marketing, Faculty of Pharmacy, “Victor Babes” University of Medicine and Pharmacy, Eftimie Murgu Square No. 2, 300041 Timisoara, Romania; 9 Department of Internal Medicine II, Faculty of Medicine, “Victor Babes” University of Medicine and Pharmacy, Eftimie Murgu Square No. 2, 300041 Timisoara, Romania

**Keywords:** betulinic acid, ursolic acid, oleanolic acid, benzotriazole esters, gold nanoparticles, melanoma, apoptosis

## Abstract

One of several promising strategies for increasing the bioavailability and therapeutic potential of high-lipophilic biologically active compounds is gold nanoparticle formulation. The current study describes the synthesis and biological antimelanoma evaluation of three triterpen-functionalized gold nanoparticles, obtained using our previously reported antimelanoma benzotriazole-triterpenic acid esters. Functionalized gold nanoparticle (GNP) formation was validated through UV-VIS and FTIR spectroscopy. The conjugate’s cytotoxic effects were investigated using HaCaT healthy keratinocytes and A375 human melanoma cells. On A375 cells, all three conjugates demonstrated dose-dependent cytotoxic activity, but no significant cytotoxic effects were observed on normal HaCaT keratinocytes. GNP-conjugates were found to be more cytotoxic than their parent compounds. After treatment with all three GNP-conjugates, 4,6′-diamidino-2-phenylindole (DAPI) staining revealed morphological changes consistent with apoptosis in A375 melanoma cells. Quantitative real-time polymerase chain reaction (RT-qPCR) analysis revealed that the triterpene-GNP conjugate treated A375 melanoma cells had a fold change increase in Bcl-2-associated X protein (BAX) expression and a fold change decrease in B-cell lymphoma 2 (Bcl-2) expression. In A735 melanoma cells, high-resolution respirometry studies revealed that all three GNP-conjugates act as selective inhibitors of mitochondrial function. Furthermore, by examining the effect on each mitochondrial respiratory rate, the results indicate that all three conjugates are capable of increasing the production of reactive oxygen species (ROS), an apoptosis trigger in cancer cells.

## 1. Introduction

Plants have represented the main source of therapeutic compounds for thousands of years, and, in the modern era, the secondary metabolites biosynthesized in plants have provided highly successful drug leads. According to the WHO, 170 member states and around 80% of the world’s population report the use of traditional medicine; in addition, over 40% of pharmaceutical formulations contain natural products, while numerous landmark drugs (i.e., aspirin, artemisinin, etc.) originated from natural sources (https://www.who.int/initiatives/who-global-centre-for-traditional-medicine; accessed on 15 September 2022). However, the world’s huge biodiversity is far from being exhausted, with many lead compounds still awaiting discovery [1]. Natural drugs exhibit several significant differences compared to synthetic molecules, such as higher molecular mass, an increased number of sp^3^—hybridized carbon and oxygen atoms combined with a decreased number of nitrogen and halogen atoms, a higher ability to form hydrogen bonds and higher hydrophilicity and molecular rigidity, all of which create an “optimized” ability to interact with biological targets [2]. Therefore, considering the growing need to find new and improved therapeutic alternatives, drug discovery has been oriented in the last few decades towards the screening of active plant constituents able to meet clinical challenges based on newly identified molecular pathophysiological findings. 

Pentacyclic triterpenes are secondary metabolites found in numerous plant species, which generally contain lupane, oleanane or ursane as main scaffolds [3]. Among them, betulinic, oleanolic and ursolic acids have been intensively studied for their anti-inflammatory, anticancer and immunomodulatory properties. However, in order to remove their disadvantages, such as poor water solubility and low oral bioavailability, a large number of semisynthetic derivatives were obtained [4]. Therefore, pentacyclic triterpenes can be used as platforms for the preparation of semisynthetic derivatives by means of various chemical processes [5]. The need for semisynthesis starting from natural drugs resides in the limitations of natural products in terms of pharmacokinetics and pharmacodynamics, which diminish their usefulness in mitigating diseases such as cancer, obesity or multiresistant infections. In addition, semisynthesis induces lower costs and poses fewer technical challenges compared to full chemical synthesis and can lead to improved pharmacological properties [6]. As for pentacyclic triterpenes, the chemical modulation of betulinic acid, for example, may be conducted in several positions on its molecule: C_3_, C_28_, C_20_–C_29_ double bond, etc.; the modulation of the C_28_ carboxyl led to various cytotoxic compounds and revealed that the carbonyl group seems to act as a pharmacophore for the cytotoxic effect [7]. The C_28_ carboxylic group can easily be functionalized through esterification, thus preserving the carbonyl moiety [8]. Consequently, ester derivatives of betulinic, oleanolic and ursolic acids are continuously developed and biologically assessed for their therapeutic potential. Among these derivatives, esters that also contain a 1,2,3-triazol ring in the C_28_ position-linked side chain proved to exhibit a higher biological potential as compared to their parent compounds [9,10,11,12]. 

In addition, in order to optimize the delivery of these types of phytocompounds and their derivatives, various approaches were employed, including their formulation as gold nanoparticle conjugates [13]. Recently, gold nanoparticles have drawn attention as nanocarriers due to their good biocompatibility and controlled distribution. Moreover, they can be obtained through simple chemical reactions, and their surfaces can be functionalized with various linkers that significantly broaden their applications in diagnosis and therapy [14]. The 1,2,3-triazol ring, which can be adsorbed onto the surface of gold nanoparticles, is one of several chemical moieties that can be used as a linker between the gold nanoparticle surface and active compounds [15]. Furthermore, because this heterocycle can be found in the structure of many triterpenic derivatives with increased therapeutic potential [12,16], the synthesis of gold nanoparticles functionalized with active triterpenic derivatives bearing 1,2,3-triazole moieties represents a promising strategy for the development of new therapeutic candidates.

Our group recently reported the synthesis and biological assessment of benzotriazole esters of betulinic, oleanolic and ursolic acids (BA-HOBt, OA-HOBt and UA-HOBt), which have shown a selective in vitro anti-melanoma effect against the A375 cell line [17]. However, these esters as well as their parent molecules are highly lipophilic structures, a characteristic that can hinder the molecule’s antiproliferative potential. Given that gold nanoparticles lack cytotoxicity, are readily available and can attach different ligands, such as triazole rings (found in the structure of our esters), the purpose of this study was to formulate the previously reported benzotriazole-triterpenic acid ethers as GNP conjugates and evaluate their antimelanoma potential to determine if these conjugates improve the biologic activity of the three triterpene derivatives.

## 2. Results

### 2.1. Synthesis and Characterization of Citrate-Capped and Triterpene-Loaded GNP

**Figure 1:**
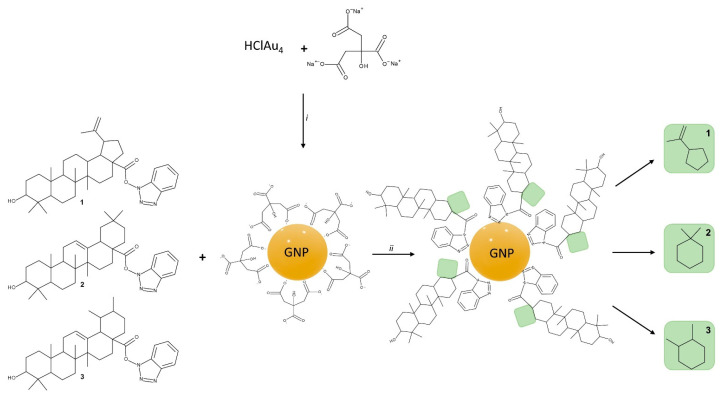
Synthesis route for citrate-capped GNP, BA-HOBt (**1**) loaded GNP, OA-HOBt-loaded GNP (**2**) and UA-HOBt-loaded GNP (**3**); conditions: i. H_2_O, boil, ii. Ultrasonication, 1 h.

Chloroauric acid was reduced with trisodium citrate in order to produce citrate-capped GNP (Figure 1). The functionalization of citrate-capped GNP with the triterpenic acid esters BA-HOBt, OA-HOBt and UA-HOBt was simply achieved by ultrasound-assisted dispersion of the triterpenic acid derivative into the GNP nanosuspension that allowed the 1,2,3-triazol moiety to attach itself to the GNP surface (Figure 1). 

The synthesis procedure used to obtain citrate-capped and triterpene-loaded GNP is depicted in Figure 1.

**Figure 2:**
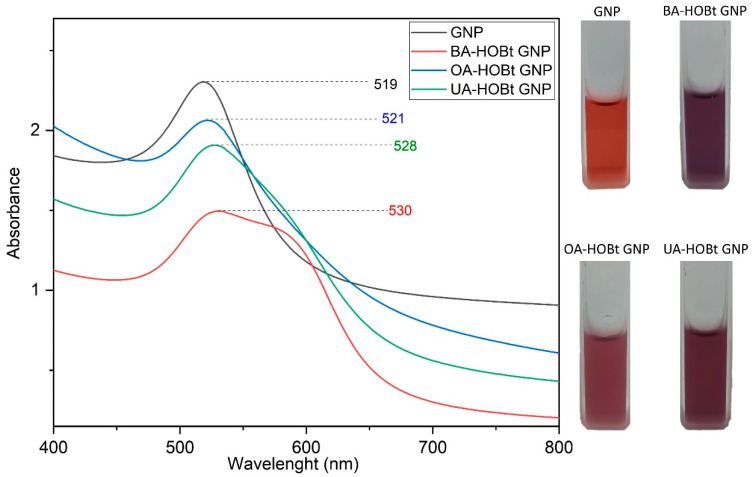
UV-VIS spectra and nanosuspension physical aspects of citrate-capped GNP, BA-HOBt GNP, OA-HOBt GNP and UA-HOBt GNP.

The synthesis and functionalization of GNP were monitored by UV-VIS spectroscopy. The analysis confirmed the formation of GNP via the citrate reduction method. GNP had the typical maximum absorbance peak at 520 nm (Figure 2). The triterpene functionalization of GNP caused the nanosuspension to change color from ruby red to purple. Red shift changes in the SPR maximum of GNP can be linked to a reduction in overall nanoparticle size, surface functionalization, or an increase in size dispersion [18,19]. The UV-VIS spectra of BA-HOBt, OA-HOBt and UA-HOBt GNP (Figure 2) show a slight red shift and a broader surface plasmon when compared to GNP. While these changes show that the triterpene functionalization reaction occurred, the spectra also show that, in addition to the surface change, an increase in particle size and dispersity or partial aggregation may have occurred in the triterpene-functionalized GNP. 

**Figure 3:**
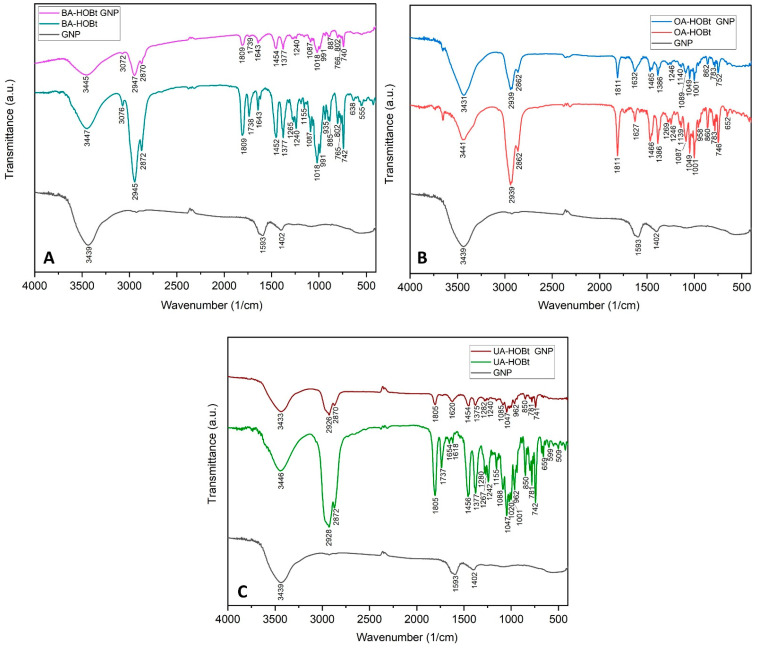
Comparative FTIR spectra of BA-HOBt GNP (**A**), OA-HOBt GNP (**B**) and UA-HOBt GNP (**C**) with GNP and the unformulated parent compound.

FTIR spectroscopy was used to validate the triterpene derivative functionalization of GNP (Figure 3). The FTIR spectra of GNP are consistent with previous data on citrate GNP [20,21] and show the two characteristic symmetric and anti-symmetric stretch vibration peaks of citrate carboxylate (1593 and 1402 cm^−1^, respectively), as well as the O-H stretch vibration at 3439 cm^−1^. The FTIR spectra of BA-HOBt GNP (Figure 3A), OA-HOBt GNP (Figure 3B) and UA-HOBt GNP (Figure 3C) clearly show that GNP was successfully functionalized by the triterpenic acid derivative in each case. The majority of BA-HOBt, OA-HOBt and UA-HOBt peaks are observable in each triterpene-functionalized GNP’s spectra. Assigned FTIR suggestive peaks of both triterpene derivatives, BA-HOBt, OA-HOBt and UA-HOBt [17], and their corresponding GNP formulations are depicted in Table 1.

**Figure 4:**
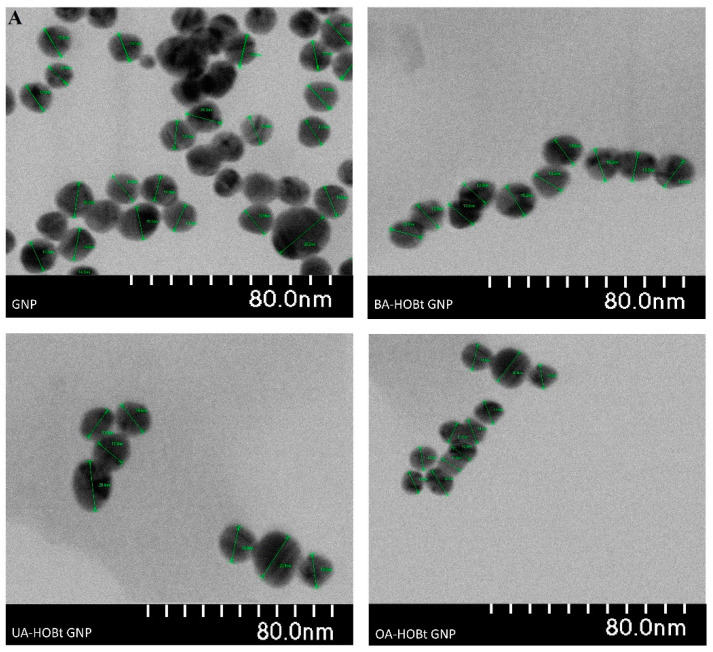

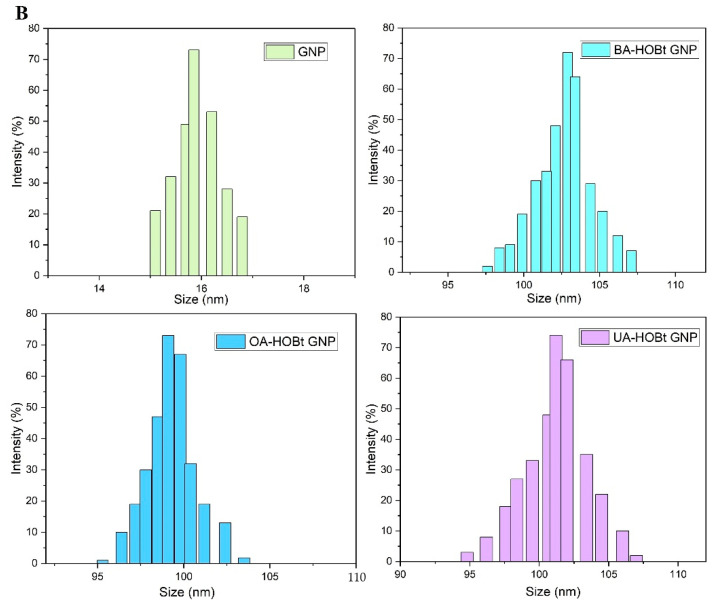
TEM images (**A**) and histograms (**B**) representing the hydrodynamic size variation of GNP BA-HOBt GNP, OA-HOBt GNP and UA-HOBt GNP.

Stable, spherical GNP nanoparticles with sizes between 13 and 25 nm were uncovered by TEM analysis (Figure 4). Triterpene functionalization of GNP had no effect on the shape or the diameter of the functionalized nanoparticles, according to TEM examination (Figure 4). However, dynamic light scattering (DLS) results demonstrated that BA-HOBt GNP, OA-HOBt GNP and UA-HOBt GNP grew in hydrodynamic size and possessed a slightly higher polydispersity index (PDI). The calculated drug loading efficiency (DLE%) was 34.2%, 30.5% and 33.8% for BA-HOBt GNP, OA-HOBt GNP and UA-HOBt GNP, respectively. The recorded ξ potential for all three types of assessed nanoparticles was roughly around −24 mV and slightly greater as compared to un-functionalized GNP, with a ξ value of −28 mV (Table 2). Even though we would normally anticipate a greater increase as a result of the elimination of negatively charged carboxilates, the slight increase may be due to the fact that not all citrate ligands were exchanged for triterpenic esters.

**Figure 5:**
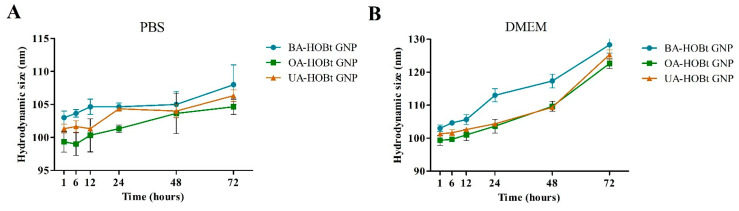
Recorded hydrodynamic sizes of BA, OA and UA conjugated GNP redispersed in PBS (**A**) and DMEM supplemented with 10% FBS (**B**), at different time points.

We recorded the UV-VIS spectra of the three nanosuspensions in water at neutral pH, phosphate buffer saline (PBS, pH 7.4) and Dulbecco’s modified Eagle Medium (DMEM, pH 7.4), which contains 10% fetal bovine serum (FBS), as this is the culture medium used for all cell experiments. During a 96-h period, we measured the full width at half maximum (FWHM) and the absorbance at maximum SPR (A_max_) at different time points. The results (Table 3) indicate that all three GNP conjugates were stable in PBS, as the FWHM and A_max_ values did not change significantly over the course of the test period. The water results were omitted because there were no recorded changes after 96 h. However, after 48 h, the FWHM and A_max_ of all three conjugates dispersed in DMEM exhibited significant changes consistent with a redshift and peak broadening, indicating an increase in dispersity. After 96 h, flocculation was observable in all three DMEM-dispersed samples. DMEM is a medium rich in amino acids that has been supplemented with protein serum. These components may also result in the displacement of surface ligands, leading to aggregation. These results are supported by DLS measurements, in which an increase in hydrodynamic size was observed in the sample dispersed in DMEM after 48 h (Figure 5). In spite of this, the formulations were shown to be stable for 48 h, which was sufficient for the biological results to be unaffected.

### 2.2. Effect of GNP and Triterpene-GNP Conjugates on Cell Viability

**Figure 6:**
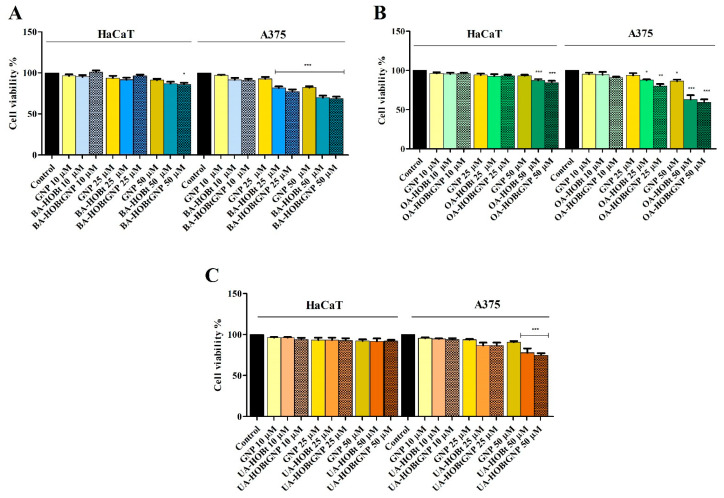
Cell viability of HaCaT and A375 cells after 24 h of treatment with 10, 25 and 50 μM of BA-HOBt GNP (**A**), OA-HOBt GNP (**B**) and UA-HOBt GNP (**C**), determined using the Alamar Blue assay. The results are expressed as cell viability percentage (%) normalized to control (100%) and are represented along with previously reported viability data for the unformulated triterpenic acid -bezotriazole esters tested in the same conditions [17], in order to evaluate statistically significant differences. The data represent the mean values ± SD of three independent experiments performed in triplicate. Statistical differences vs. control were determined using one-way ANOVA analysis followed by Bonferroni multiple comparisons post-test (* *p* < 0.05, ** *p* < 0.01 and *** *p* < 0.001).

In order to verify if BA-HOBt GNP, OA-HOBt GNP, UA-HOBt GNP and GNP alone are able to influence the cellular viability of healthy human keratinocytes HaCaT and of human melanoma A375 cells, the Alamar Blue assay was employed. As presented in Figure 5, only the highest concentrations of BA-HOBt GNP and OA-HOBt GNP exhibited low toxicity on normal cells (BA-HOBt GNP: 82.6% HaCaT viable cells and OA-HOBt GNP: 84.7% HaCaT viable cells), whereas UA-HOBt GNP did not influence the cellular viability of normal cells at any of the concentrations tested. Upon testing on A375 melanoma cells, all three compounds elicited a significant decrease in cell viability vs. control at 25 and 50 μΜ concentrations (Figure 6). In detail, BA-HOBt GNP decreased cell viability at 77.2% when tested at 25 μΜ and at 69% when tested at 50 μΜ, OA-HOBt GNP decreased cell viability at 81.2% (25 μΜ) and 59.3% (50 μΜ), while UA-HOBt GNP influenced cell viability to a lesser extent compared with the previous nanoformulations, producing only a decrease to 86,8% (25 μΜ) and to 74.8% (50 μΜ) of the A375 cell line (Figure 6). The viability results of GNP formulations were displayed alongside the cytotoxic activity of the previously described benzotriazole-triterpene acid esters [17]. While GNP-conjugates exhibit a slight decrease in cell viability compared to their unformulated counterparts, the only significant difference in cytotoxicity between GNP-conjugated and unformulated triterpenic acid was observed when OA-HOBt GNP was tested at 25 μM.

### 2.3. Morphological Assessment of Apoptotic Cells by Microscopic Evaluation and DAPI Staining

**Figure 7:**
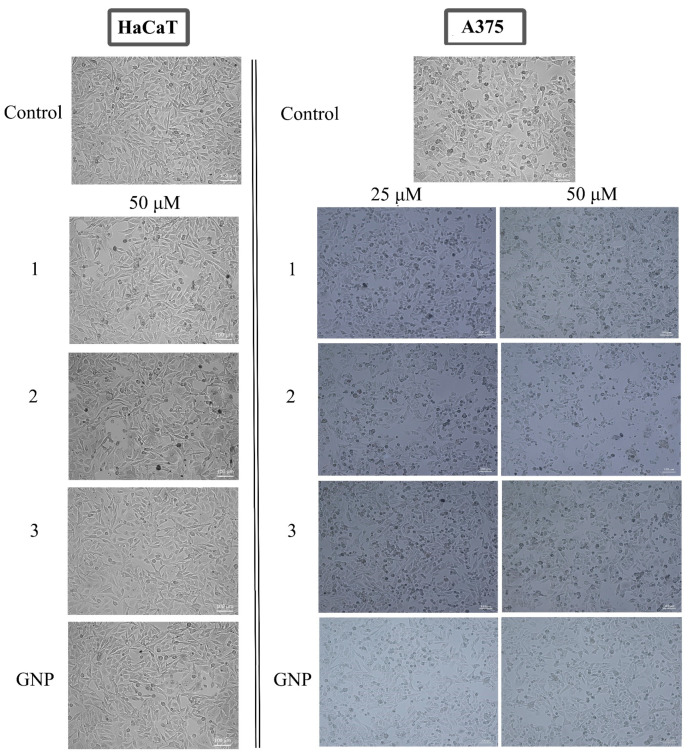
Representative images of the morphological aspect of HaCaT (50 μM) and A375 (25, 50 μM) cells after treatment for 24 h with BA-HOBt GNP (**1**), OA-HOBt GNP (**2**) and UA-HOBt GNP (**3**). The scale bar was 100 μm.

In order to highlight the underlying mechanism of action, the assessment of cell morphology was conducted by microscopic evaluation on both cell lines; the cytotoxic effect is indicated by changes in the cells’ morphological features such as shape, adherence and confluence. Examining the A375 cells, one could notice the occurrence of round cells with reduced intercellular adherence and also reduced adherence to the plaque accompanied by low confluence, features indicating cytotoxic activity. All these aspects showed a dose-dependent manner of increase. Similar changes were recorded in HaCaT cells as well, but to a much lower extent (Figure 7).

**Figure 8:**
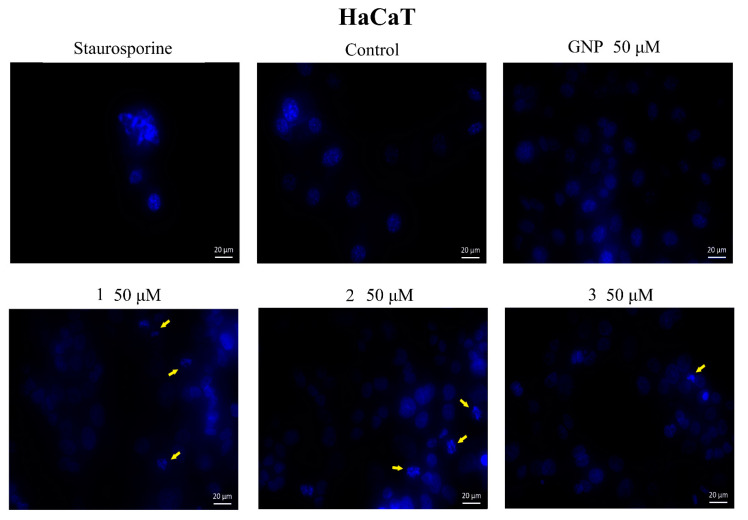
Nuclear staining using DAPI in HaCaT cells after treatment with GNP (50 μΜ), BA-HOBt GNP (**1**), OA-HOBt GNP (**2**) and UA-HOBt GNP (**3**) (50 μM) for 24 h. The pictures were captured 24 h post-treatment. The staurosporine solution (5 μM) was used as a positive control for apoptotic changes at the nuclear level. The yellow arrows represent signs of apoptosis such as nuclear shrinkage, condensation, fragmentation and cellular membrane disruption.

**Figure 9:**
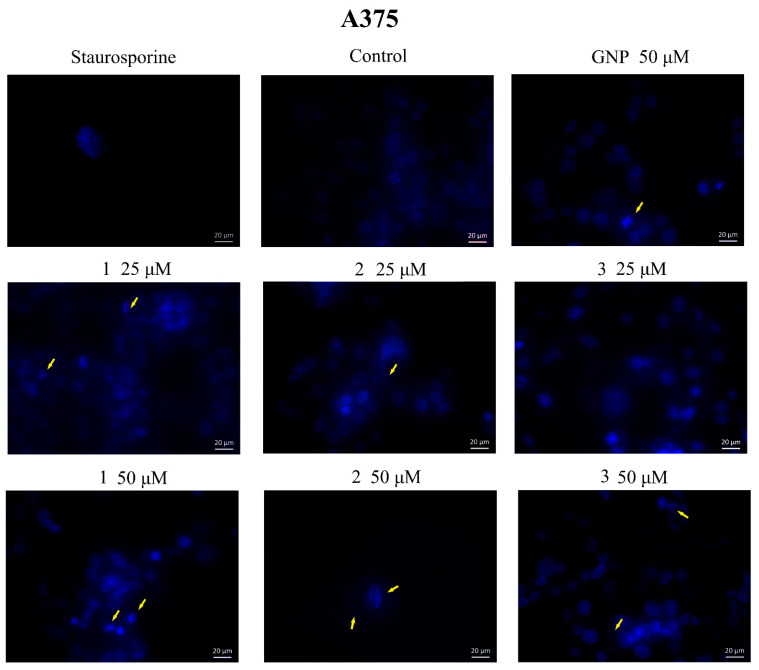
Nuclear staining using DAPI in A375 cells after treatment with GNP (25, 50 μΜ), BA-HOBt GNP (**1**), OA-HOBt GNP (**2**) and UA-HOBt GNP (**3**) (25 and 50 μM) for 24 h. The pictures were captured 24 h post-treatment. The staurosporine solution (5 μM) was used as a positive control for apoptotic changes at the nuclear level. The yellow arrows represent signs of apoptosis such as nuclear shrinkage, condensation, fragmentation and cellular membrane disruption.

In addition, cells were investigated by a DAPI test, which consists of nuclear staining with a 4,6′-diamidino-2-phenylindole (DAPI) reagent. Results are depicted in Figure 8 and Figure 9. Nuclear condensation, shrinkage and fragmentation (yellow arrows) were not so frequent in HaCaT cells (Figure 8) but were significantly observed in A375 cells treated with the highest two tested concentrations (25, 50 μM) of BA-HOBt GNP, OA-HOBt GNP and UA-HOBt GNP, with the most visible effect being recorded at 50 μM, in all three cases (Figure 8). According to these findings, triterpene-GNP conjugates in high concentrations (25, 50 μM) exhibit cytotoxic effects on A375 melanoma cells, significantly decreasing cell viability and inducing morphological apoptotic features.

### 2.4. High-Resolution Respirometry Studies

**Figure 10:**
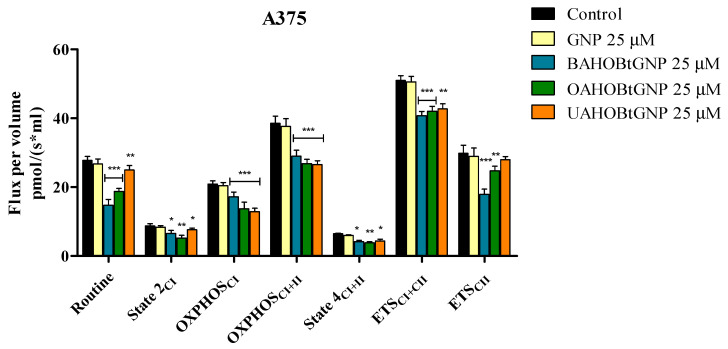
Respiration of permeabilized human melanoma cells (A375) following 24 h stimulation with 25 μM GNP, BA-HOBt GNP, OA-HOBt GNP and UA-HOBt GNP. Data represent the mean ± SD of five individual experiments. Values with *p* < 0.05 were considered have a statistically significant difference (* *p* < 0.05, ** *p* < 0.01 and *** *p* < 0.001). The respiratory parameters displayed represent the following: Routine—respiration of cells suspended in a substrate-free media, supported by endogenous ADP; State 2_CI_—mitochondrial respiration in basal conditions driven by CI; OXPHOS_CI_—active respiration dependent on CI substrates and exogenous ADP; OXPHOS_CI+II_—maximal active respiration driven by both CI and CII; State 4_CI+II_—LEAK respiration dependent on both CI and CII; ETS_CI+II_—maximal respiratory capacity of the electron transport system in the fully noncoupled state; ETS_CII_—electron transport system maximal capacity dependent only on CII.

Normal HaCaT and A375 melanoma cell lines were treated with three GNP-conjugated benzotriazolyl esters of betulinic, oleanolic and ursolic acids for 24 h and tested by means of high-resolution respirometry. The compounds did not significantly influence the mitochondrial respiration of normal HaCaT cell lines at 25 μM. A major finding of this work is that each new conjugate can inhibit the mitochondrial respiration of A375 melanoma cells when tested at 25 μΜ. In detail, control and treated cells were suspended in mitochondrial respiration media (MIRO5) and were allowed to respire on the existing substrates from the media and on the endogenous ones for 15 min. In this physiological routine state, respiration and phosphorylation are controlled by energy turnover and demand and by the level of intrinsic uncoupling or dyscoupling [22]. All three tested conjugates decreased routine respiration rate vs. control, as follows: 14.72 vs. 24.23 for BA-HOBt GNP, 18.79 vs. 28.44 for OA-HOBt GNP and 24.97 vs. 31.13 for UA-HOBt GNP (Figure 10). The following addition of digitonine and CI substrates decreased mitochondrial respiration to State2_CI_, the basal respiration. When compared to controls, cells treated with all three conjugates presented a significantly decreased State2_CI_, whereas cells that received treatment with GNP alone did not present this decrease in State2_CI_. Upon analyzing the active respiration (OXPHOS_CI_ and OXPHOS_CII_), a decrease in both respiratory rates vs. control was observed after treatment with BA-HOBt GNP (17.18 and 28.97 vs. 23.44 and 44.17), OA-HOBt GNP (13.74 and 26.83 vs. 18.76 and 34.06) and UA-HOBt GNP (12.86 and 26.53 vs. 20.38 and 37.44) (Figure 9). Moreover, all three conjugates produced a similar inhibitory effect on the maximal respiratory electron-transfer pathway capacity (ETS_CI+CII_ and ETS_CII_) vs. control, as follows: 40.7 and 17.09 vs. 54.88 and 23.61 for BA-HOBt GNP, 42.04 and 24.71 vs. 48.59 and 30.96 for OA-HOBt GNP and 42.71 and 27.98 vs. 49.38 and 34.75 for UA-HOBt GNP (Figure 9). The respiration compensating for the proton flux across the inner mitochondrial membrane, bypassing the ATP synthase (proton leak, State4_CI+CII_) was also decreased as a result of functionalized GNP treatment vs. control cells (for the BA conjugate: 4.17 vs. 6.2; for the OA conjugate: 3.84 vs. 6.75 and for the UA conjugate: 4.37 vs. 6.54). Bare GNP was not able to modify in a statistically significant manner vs. control any of the mitochondrial rates measured (Figure 9). 

### 2.5. Quantitative Real-Time PCR (RT-qPCR)

**Figure 11:**
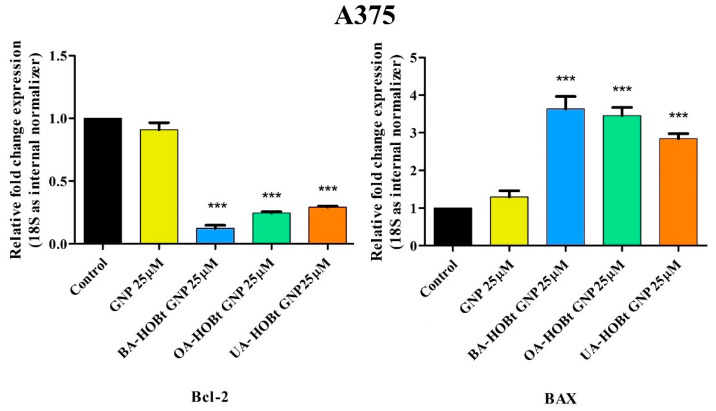
Relative fold change expression of *Bcl-2* and *BAX* mRNA in A375 cells after 24 h stimulation with GNP, BA-HOBt GNP, OA-HOBt GNP and UA-HOBt GNP (25 μM). The expressions were normalized to 18S, while the control values were obtained from experiments with untreated cells. The data represent the mean ± standard deviation of three independent experiments. The statistical differences between control vs. stimulated cells were determined using one-way ANOVA with Dunnett’s post-test (*** *p* < 0.001).

Quantitative real-time PCR was used to determine gene expression variations of anti-apoptotic Bcl-2 and pro-apoptotic BAX to further establish the pro-apoptotic effects of the three triterpene-gold nanoformulations on A375 melanoma cells. After 24 h of incubation, measurements were taken on A375 cells treated with test nanoconjugates. Each conjugate’s effect was measured at the lowest concentration at which it caused a significant reduction in cell viability (25 μM). The results show that all three conjugates increase the expression of the pro-apoptotic *BAX* gene, whereas GNP had not produced any significant changes in fold expression. In the case of the anti-apoptotic *Bcl-2* gene, while all triterpene-functionalized GNP decreased the expression, no significant changes were observed for the un-functionalized GNP (Figure 11).

### 2.6. Effect of GNP and GNP-Conjugated Benzotriazolyl Esters of Betulinic, Oleanolic and Ursolic Acids on Key Pro and Antiapoptotic Markers

**Figure 12:**
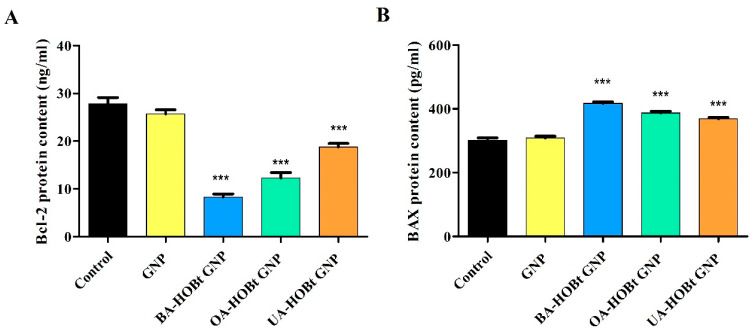
Effect of GNP, BA-HOBt GNP, OA-HOBt GNP and UA-HOBt GNP on the Bcl-2 (**A**) and Bax (**B**) protein content in A375 cancer cells treated with the compounds at 25 μM. Data are expressed as mean values ± SD. The statistical differences between control vs. stimulated cells were determined using one-way ANOVA with Dunnett’s post-test (*** *p* < 0.001).

Cell apoptosis is a complex biological process that is regulated via an intricate signaling pathway, including here the Bcl-2 family of proteins that regulate the intrinsic apoptosis pathway at the mitochondrion level [23]. In the present study, treatment of A375 cells with 25 μM of BA-HOBt GNP, OA-HOBt GNP and UA-HOBt GNP resulted in a significant decrease of Bcl-2 protein content and an increase of BAX protein content compared to the control (Figure 12A,B). In detail, BA-HOBt GNP, OA-HOBt GNP and UA-HOBt GNP decreased the mean values of Bcl-2 to 8.39, 12.27 and 18.79 ng/mL vs. control (27.86 ng/mL); BAX was increased as follows: 417.42, 387.25 and 368.9 vs. control (302.58 ng/mL). Treatment with bare GNP was not able to induce any statistical difference vs. control in either Bcl-2 or BAX protein content (Figure 12). 

## 3. Discussion

Triterpenic acids, betulinic, oleanolic and ursolic acids, have become subjects of interest in cancer research ever since betulinic acid was identified as a highly selective anti-melanoma agent in 1995 by Pisha et al. [24]. They exhibit strong anticancer properties [25] but poor pharmacokinetic profiles, presumably due to their high lipophilicity [26,27]. Overcoming this issue was possible through various nanoformulations [28] but also through chemical modulation [5]. Our group has previously designed and synthesized benzotriazole esters of betulinic, oleanolic and ursolic acids, respectively. When tested in A375 and HaCaT cells, all three compounds proved cytotoxic against melanoma cells, with the UA derivative displaying lower effects compared to the other two. However, no signs of toxicity against normal HaCaT cells were noticed for either compound, thus revealing selective antitumor properties. Molecular docking, confirmed through biological assessment, showed the significant affinity of all three esters for the anti-apoptotic Bcl2 protein, thus indicating their apoptotic anti-melanoma effects [17]. 

In the current study, in order to further improve the anticancer properties of the benzotriazole esters, we employed gold nanoparticles as carriers. We previously successfully synthesized betulin-coated GNPs that proved to be efficient delivery systems for the loaded phytocompound, with the resulting nanoformulation acting as a cytotoxic and apoptotic agent in a dose- and time-dependent manner [29]. We used the same, slightly modified Turkevitch method for the synthesis of GNP, which involves sodium citrate as both a reducing agent and stabilizer. Spherical nanoparticles were obtained with diameters ranging from 13 to 25 nm, which is consistent with previously reported results [30,31]. The conjugation of the benzotriazole esters onto the metallic gold surface was possible through the benzotriazole moiety, which has the ability to coordinate the Au atoms and also to delay nanoparticles’ agglomeration [32]. Ultrasound irradiation is frequently employed in the sonochemical synthesis of GNP, where parameters like frequency, power or exposure time have various influences on the different physical characteristics of the obtained nanoparticles [33]. For this step, ultrasound irradiation (200 W) was used for the functionalization step that both reduced the reaction time and increased the dispersion of the hydrophobic triterpene derivatives into the aqueous medium. Formation of the triterpene-functionalized GNP was indicated by the color change and the slight red shift recorded by UV-VIS spectroscopy and FTIR analysis. The FTIR spectra of triterpene-functionalized GNP clearly show that the majority of assigned peaks have a corresponding value in the FTIR spectra of the unformulated triterpenic acid esters. The red shift and broadening of the surface plasmon of triterpene-functionalized GNP, as previously stated, can be attributed to surface functionalization, size increase or dispersity [19]. Subsequent analysis, such as TEM, revealed that the functionalization had no effect on the mean size. According to the literature, ultrasound irradiation at high power levels (up to 210 W) induces the formation of smaller and more spherical GNP [34]. These findings are also supported by a previously reported sonochemical synthesis of functionalized GNP [33]. Oladimeji et al. reported a similar study on the synthesis of BA-functionalized GNP, where BA functionalization was not conducted using ultrasonic irradiation, but this process did not alter the spherical shape or size as shown by TEM analysis [13]. Overall, ultrasonic radiation may have had a significant effect on keeping the nanoparticles small and spherical, but it was not the sole cause of these results. DLS measurements were recorded in aqueous media at pH 7. The functionalization process led to an increase in hydrodynamic size and polydispersity index for all samples. These parameters can also induce the red shift in the SPR maximum of functionalized GNP and are consistent with previously reported triterpene and other types of functionalized GNP [13,35,36]. The ζ potential of colloidal systems can be used as a predictor for the stability of the analyzed nanosuspension. While higher absolute values of ζ potential indicate a more stable state of the nanosuspension, potential values greater than +30 mV or less than −30 mV allow for an essentially stable colloidal system [37]. The recorded ζ potentials in our case were similar for all tested samples (−28.2 mV for GNP vs. −23.2 for BA-HOBt GNP, −24.0 for OA-HOBt GNP and −23.2 for UA-HOBt GNP respectively). These values would suggest that triterpene-functionalized GNP would have lower stability as compared to unfunctionalized GNP but are, at the same time, consistent with stable GNP with a low probability of forming aggregates over time [38]. A recent study reported the stability of GNP in aqueous media with similar ζ values for a period of 60 days, during which no significant changes were recorded in the conducted measurements [39]. DLS results are consistent with the previously cited study of Oladimeji et al., which showed that GNP functionalization with BA, by using epigallocatechin gallate as a linker, also led to an increase in hydrodynamic size, dispersity and ζ potential [13]. Sadly enough, no other available literature regarding the stability of any triterpene-functionalized GNP is available. The three triterpene-GNP conjugates were stable for 96 h in water and PBS, but their stability began to decline after 48 h when resuspended in DMEM. This is consistent with other reports in which various surface-modified GNPs demonstrated a decrease in stability when resuspended in DMEM, accompanied in some cases by a redshift in the UV-VIS spectra [40,41]. Even in the case of highly stable dendron-modified GNP, whose stability was only affected by redispersion in DMEM, this pattern was observed [42]. As these reports show, one major contributor to the decrease in colloidal stability is due to the protein-ligand exchange at the surface of GNP. While these types of GNP with different surfaces (no ester bonds) demonstrated a decrease in stability around the same time, and given the high reactivity of our surface ligands, it is also plausible to hypothesize that the GNPs may provide triterpenic esters with a prolonged protection effect against hydrolysis.

The conjugated GNP was further tested against the same A375 melanoma cell line as well as in normal HaCaT cells in order to assess their cytotoxic effects and antitumor selectivity by using the Alamar Blue assay; naked GNP was used as a reference. The Alamar Blue test is widely used for quantitative assessment of cell viability, migration and proliferation. Alamar Blue is an indigo-colored, water-soluble, non-toxic reagent able to detect metabolically active cells [43]. As a result of cell mitochondrial enzymatic activity, the reagent changes from indigo to fluorescent pink, which can be easily quantified spectrophotometrically. All three conjugates displayed cytotoxic activities in a dose-dependent manner; thus, in A375 melanoma cells, 25 μΜ of conjugates 1, 2 and 3 induced 77,2%, 81.2%, and 86.8%, respectively, decreases in cell viability, while the highest concentration (50 μΜ) caused a more significant effect, with cell viability reaching 69%, 59.3% and 74.8% for conjugates 1, 2 and 3, respectively. One can notice that, compared to the esters alone, where higher cell viability percentages were recorded [17], the cytotoxic effects of the respective GNP-conjugates were slightly improved, thus indicating that the metallic nanocarrier to some extent promoted the compounds’ cytotoxicity. In addition, the three conjugates were applied to normal HaCaT cells, where low toxicity was reported only when the higher concentration was used: 82.6% and 84.7% viable cells for conjugates 1 and 2, respectively. No signs of toxicity occurred for conjugate 3 in either concentration or for conjugates 1 and 2 when applied at 25 μΜ. Of note, bare GNP does not induce cytotoxic effects in either tumor or healthy cells. These findings emphasize a similar behavior with the three esters used alone that also proved non-toxic in small concentrations, diminishing cell viability only when used in higher concentrations; the ursolic acid derivative as well as its GNP conjugate lacked cytotoxicity in HaCaT cells regardless of their concentrations, thus indicating safe application in cancer treatment [17]. The literature reports gold nanoparticles as drug carriers in cancer cells; as an example, the flavonoid hesperidine was loaded on GNP through electrostatic interactions in a similar manner to the one described in the current study but using sodium borohydride as a reducing agent [35]. The loaded GNP exhibited stronger cytotoxic effects than both hesperidin and GNP alone and in lower concentrations, exerted in a dose-dependent manner; the authors hypothesized that the reduction in cell viability in direct proportion with an increased nanoparticle concentration indicates a higher accumulation of nanostructures inside tumor cells, leading to cell death. The penetration of gold nanoparticles in certain cells, such as white blood cells, was already reported [44]. Depending on their shape, size and surface chemistry, most GNPs are able to penetrate cells in various amounts [45,46,47,48], where some chemical functional groups, such as the ester group, seem to increase the GNP cell penetration. This hypothesis can also be formulated for the currently reported results; however, further studies are needed in order to validate this hypothesis. The literature is rather scarce in terms of studies focused on triterpene-conjugated GNP. To the best of our knowledge, only betulin was loaded on GNP through electrostatic interactions as a result of the Turkevitch method that was also applied in the current study.

The underlying mechanism of anticancer activity reported for triterpenic acids consists of apoptosis induction [26,27]; also, we previously reported an apoptotic mechanism for the benzotriazole esters of the three triterpenic acids, betulinic, oleanolic and ursolic [17]. It is therefore presumable that the conjugated esters will exert similar mechanisms by regulating the expression of several pro- and antiapoptotic proteins. Apoptosis is a process whose induction is frequently encountered in anticancer mechanisms [49], which involves programmed cell death and plays a crucial role in homeostasis and cancer prevention [50]. The first step towards the identification of the mechanism of action for the three GNP conjugates was the morphological assessment of treated cells, both malignant and healthy. The results showed round, floating cells with low confluence and adherence, parameters that indicate cytotoxic effects. Similar results were reported for the three esters alone [17]; spherical gold nanoparticles exerted very low cytotoxic effects in cancer cells [51], so we may assume that the cytotoxic effect is given by the presence of the semisynthetic triterpenes.

In order to identify a potential apoptotic mechanism, both types of cells were submitted to DAPI staining, which highlights nuclear changes by blue fluorescence under the UV light; HaCaT cells exhibited moderate signs of apoptosis for the higher concentrations of BA-HOBt GNP and OA-HOBt GNP, while UA-HOBt GNP completely lacked apoptotic or necrotic activity, in agreement with the previously published data on their non-conjugated counterparts [17]. In A375 melanoma cells, both concentrations caused apoptotic changes such as nuclear shrinkage and fragmentation accompanied by chromatin condensation; staurosporine was used as a positive control. These apoptotic nuclear changes were identified for numerous pentacyclic triterpenes [52] when testing the same concentrations proved efficient in viability tests against tumor cells. Naked GNP did not induce apoptotic changes in either type of cell; by contrast, Chakraborty et al. reported an apoptotic effect of citrate-coated gold nanoparticles in osteosarcoma cells [53]. However, the apoptotic effect was size-dependent, with significantly higher percentages of cell death being reported for nanoparticles ranging between 46 and 60 nm that were able to disrupt the cell’s mitochondrial membrane potential. Therefore, we may assume that the lack of apoptotic effects on bare GNP in the current study is due to their size, which varies from 13 to 25 nm, a considerably smaller diameter compared to the mentioned study. Although triterpenes are able to alter cell membrane integrity in particular when they contain glycoside moieties [54], this mechanism manifests equally on tumor and healthy cells alike, which was not the case in the current study, where normal cells were minimally affected. 

Apoptosis, or programmed cell death, is a crucial process in both physiological and pathological conditions, and understanding the underlying mechanism can offer treatment strategies in various diseases, including cancer [55]. Cancer cells are recognized for their ability to evade apoptosis and undergo numerous cell divisions with enhanced frequency. Defects in the two apoptotic pathways—the extrinsic (mediated by the death receptor) and intrinsic (mitochondrial) pathway are responsible for malignant transformation, metastasis and treatment resistance. Cell fate can therefore be dictated by mitochondrial-induced apoptosis and more specifically by the balance between the pro- and anti-apoptotic proteins of the Bcl-2 family. Thus, pharmacological interventions designed to correct any mishaps in the apoptotic pathways are considered promising anticancer agents. In our recent work, we showed that bezotriazole esters of BA, OA and UA, respectively, did reduce the expression of the anti-apoptotic Bcl-2 gene and induced an increase in the fold gene expression of the pro-apoptotic BAX in A375 melanoma cells [17]. In the present study, the same changes were reported for the three triterpenic acid esters formulated as GNP, but the difference in fold expression vs. control is higher than in the case of the unformulated triterpenic acid esters. This is especially visible in the case of UA-HOBt GNP, where the same significant changes in fold expression occurred at 25 μM, whereas the unformulated compound showed significant changes in fold expression only when tested at 50 μM. Given these facts, it is safe to assume that the GNP formulation of the benzotriazole triterpenic acid esters boosted their proapoptotic activity on A375 melanoma cells. 

The enzyme-linked immunosorbent assay (ELISA) was also used to determine the fold change in BAX/Bcl-2 protein content in A375 cells treated with triterpene conjugated GNPs. Our findings show that all three gold-based formulations increase BAX content while decreasing levels of anti-apoptotic Bcl-2. Numerous studies have shown that BA, OA, and UA cause significant fold changes in the BAX/Bcl-2 expression ratio in various cancer cells [56,57,58,59]. Furthermore, similar BA phenyl-1,2,3-triazole derivatives and UA imidazole derivatives were shown to induce the same above-mentioned protein-targeted expression fold changes in various cancer cells [60,61]. Our results are to be expected in light of these previously mentioned findings. However, while pentacyclic N containing heterocycle derivatizations of triterpenic acids does not seem to affect the Bcl-2/BAX targeted pro-apoptotic effect, the scarcity of literature on the apoptotic assessment of triterpene GNP conjugates renders further discussion impossible.

The effect of the new esters formulated with GNP on mitochondrial function was also tested using high-resolution respirometry. The obtained results demonstrated the ability of each new compound formulation to inhibit mitochondrial respiration when tested at 25 μΜ, a concentration at which the compounds produced a cytotoxic effect on A375 melanoma cell lines without showing toxic effects on normal HaCaT cells. In detail, the results show us that in A375 melanoma cells, all the compounds decreased the basal respiration (routine state), a fact that reveals their ability to decrease mitochondrial demand for ATP and energy turnover. Moreover, the triterpene GNP conjugates significantly reduced the oxidative phosphorylation capacity (active respiration—OXPHOS_CI_ and OXPHOS_CI+CII_) and the maximum capacity of the electron transport system (ETS_CI+CII_ and ETS_CII_), meaning that they all induce mitochondrial dysfunction. Another effect observed at the mitochondrial level following the treatment of cells with all three conjugates was a decrease in basal and LEAK respiration (State2_CI_ and State4_CI+CII_). This indicates that the compounds produce a decrease in oxygen consumption in the resting state, when ATP synthase is not active, and also a decrease in proton transport across the inner mitochondrial membrane. According to literature data, this effect is the opposite of the phenomenon of mitochondrial uncoupling, characterized by increased proton transport across the inner mitochondrial membrane [62]. 

**Figure 13:**
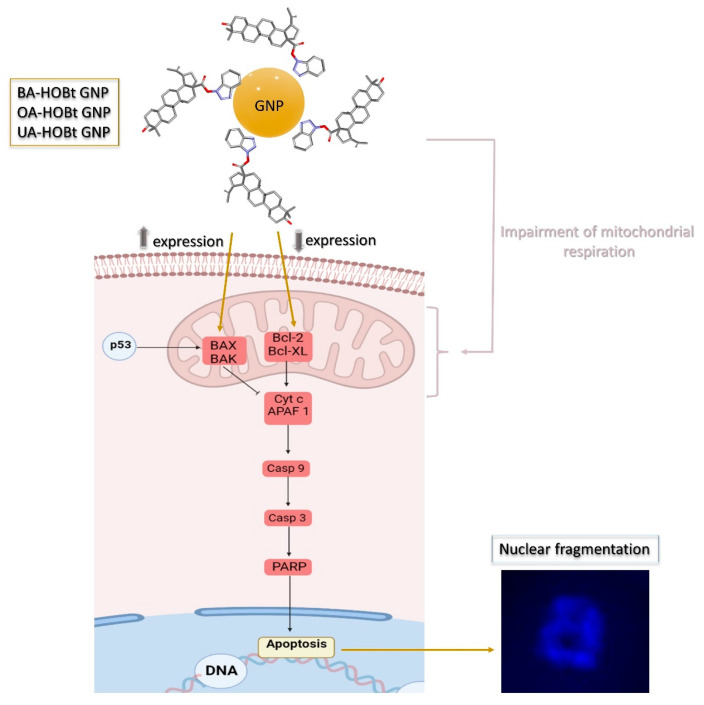
Schematic representation of the pro-apoptotic effect mechanisms highlighted by the triterpene conjugated GNP.

All these findings converge on the fact that our three formulations are able to induce apoptosis in melanoma cancer cells. While apoptosis can be triggered by multiple pathways, one mode that correlates with the triterpene-functionalized GNP’s pro-apoptotic features revolves around mitochondrial respiration inhibition, the decrease of pro-apoptotic Bcl-2 and the increase of anti-apoptotic BAX. All these findings are graphically represented in Figure 13. 

## 4. Materials and Methods 

### 4.1. Synthesis of Citrate-Capped and Triterpene-Loaded Gold Nanoparticles

Citrate-capped gold nanoparticles (GNP) were produced by employing trisodium citrate to reduce chloroaurate. Specifically, 68 mg (0.2 mmoles) of chloroauric acid (HauCl4, Merck KGaA, Darmstadt, Germany) is dissolved in 180 mL of deionized water with constant stirring. After bringing the solution to a boil, a 20-mL solution of sodium citrate dihydrate (Merck KGaA, Darmstadt, Germany) containing 176.5 mg (0.6 mmoles) is added. When the solution’s color turns ruby red, the heat is turned off and it is allowed to cool to room temperature for 24 h. UV-VIS spectroscopy confirmed the production of GNP. GNP was purified using successive centrifugations at 13,000× *g*, water removal and resuspension in water until a neutral pH value was reached.

Triterpene derivative-loaded GNP was obtained using the following method: 0.01 mmoles of benzotriazolyl esters of betulinic acid (BA-HOBt), oleanolic acid (OA-HOBt) and ursolic acid (UA-HOBt), respectively, were each added to 10 mL of previously obtained citrate-capped GNP nanosuspension. The suspension was sonicated for 1 h (0.8 cycles, 80% amplitude, 200 W) with a UP200S ultrasonic homogenizer (Hielscher Ultrasonics GmbH, Germany, Teltow) outfitted with a volume-appropriate sonotrode. After 10 min, all three samples began to turn purple, and by the end of the sonication cycle, all three samples were completely purple, indicating that GNP surface functionalization has occurred. The samples were kept at room temperature in the dark for 24 h. All three samples were later extracted with ethyl acetate (4 × 5 mL) (Merck KgaA, Darmstadt, Germany) in order to remove potentially unattached triterpene derivatives, after which each sample was purified using successive centrifugations at 13,000× *g*, water removal and resuspension in water (three cycles) until all three samples reached a neutral pH. 

### 4.2. Gold Nanoparticles Characterization 

UV-VIS spectrometry was used to monitor the formation of both citrate-capped and triterpene-loaded GNP. The samples’ adsorption spectra were measured with a Shimadzu UV-1900i spectrophotometer (Shimadzu Scientific Instruments Inc., Columbia, MD, USA) over a wavelength range of 400–800 nm. Bond formation and functionalization of triterpene-loaded GNP were determined using Fourier Transform Infrared spectrometry (FTIR). Aliquots of a 3 mL GNP suspension were centrifuged at 13,000× *g* and then oven-dried at 80 °C for 24 h to produce dried GNP samples. All spectra were recorded on a Shimadzu IR Affinity-1S spectrophotometer (Shimadzu Scientific Instruments Inc., Columbia, MD, USA) in the range of 400–4000 cm^−1^ (4 cm^−1^ resolution) using potassium bromide pellets. The particle size was evaluated by transmission electron microscopy (TEM) with a Hitachi HD2700 cold field emission gun STEM (Chiyoda, Tokyo, Japan) fitted with two windowless EDX detectors (X-MaxN 100). The Vasco Particle Size Analyzer and Wallis Zeta Potential analyzer (Cordouan Technologies, Pessac, France) were used to measure the size and stability of the aqueous solutions (at neutral pH) in the samples that were studied. For data acquisition, the following size analyzer parameters were preset: DTC position down, temperature 25 °C, 90% laser power, number of channels 339, time interval 16 milliseconds, acquisition mode continuous, analysis mode cumulants; and for the potential analyzer: 25 °C, 80% laser power, plastic cuvette, 5 electrode distance, medium resolution and Henry function Huckel. Over a 96-h period, UV-VIS and DLS measurements were taken to evaluate the triterpene-conjugated GNP’s stability. Experiments were conducted in neutral pH water, PBS (at pH 7.4) and DMEM containing 10% FBS (at pH 7.4). All experiments were conducted at 37 °C. The maximum absorbance (A_max_) and calculated full width at half medium (FWMH), derived from the recorded UV-VIS spectra, along with the average hydrodynamic size, were used as stability indicators.

### 4.3. Drug Loading Efficiency Determination

The drug loading efficiency of triterpene-GNP nanoformulations was determined using LC-MS spectroscopy on a 6120 LC-MS analytical system (Agilent, Santa Clara, CA, USA) consisting of a 1260 Infinity HPLC equipped with a G1322A degasser, a G1311B quaternary pump, a G1316A column thermostat, a G1365C MWD detector and a G1328C manual injector coupled with a Quadrupolar (Q) mass spectrometer equipped with electrospray ionization source (ESI). To hydrolyze triterpenic acids (BA, OA and UA) from HOBt, which acted as a linker between the triterpenic acid and GNP, a 10 mL aliquot of each sample was treated with NaOH 4 M for 24 h. The suspension was neutralized with concentrated HCl before being extracted with ethyl acetate. After removing the solvent from the organic fractions, the residue was redissolved in methanol. The concentrations of BA, OA and UA, respectively, were determined using a 7-point plot curve, as described previously [63]. The following formula was used to calculate drug loading efficiency:

(1) $$\mathrm{DLE}\%=\frac{\mathrm{We}}{\mathrm{Wt}}\times 100$$

where

We = quantity of the encapsulated drug, spectrometrically determined.
Wt = quantity of total drug added to the nanoformulation.

### 4.4. Cell Culture 

The cells, immortalized human keratinocytes—HaCaT (CLS Cell Lines Service GmbH, Eppelheim, Germany) and human melanoma cell lines—A375 (ATCC^®^ CRL-1619™, American Type Culture Collection-ATTC, Lomianki, Poland), were received as frozen items and were stored in liquid nitrogen. Both cell lines were cultured in Dulbecco’s modified Eagle Medium (DMEM) with high glucose, supplied with a mixture of 10% fetal bovine serum (FBS) and 1% a combination of 100 U/mL penicillin and 100 U/mL streptomycin (Sigma-Aldrich, Munich, Germany) and maintained in a humidified incubator with 5% CO_2_ at 37 °C. After reaching 85–90% confluence, cells were stimulated with the tested triterpene GNP nanoformulations and GNP (10, 25 and 50 μΜ) for 24 h. The cell number was determined in the presence of Trypan blue using an automated cell counting device (Thermo Fisher Scientific, Inc., Waltham, MA, USA).

### 4.5. Cell Viability Assessment—Alamar Blue Assay and Cell Morphology

Alamar Blue Staining. The cytotoxic activity of GNP and triterpene GNP conjugates (10, 25 and 50 μΜ) on HaCaT and A375 cell lines was assessed by means of the Alamar Blue assay. The cells (1 × 10^4^ cells/well) were cultured on a 96-well plate and allowed to attach for 24 h, while being incubated at 37 °C and 5% CO_2_. The next day, the culture medium was replaced with fresh medium, and the cells were stimulated with the tested compounds. After 24 h, the cells were further counterstained with Alamar Blue 0.01% (20 μL working solution) and incubated again for 3 h at 37 °C. To quantify the cell viability, the absorbance was measured at 570 and 600 nm using a microplate reader (xMark™ Microplate Spectrophotometer, Biorad, Hercules, CA, USA).

Cellular morphology evaluation. The effect of GNP and triterpene GNP conjugates (10, 25 and 50 μΜ) on cellular morphology was assessed immediately after the addition to compounds on freshly attached cells (0 h) and at 24 h post-treatment. The images were captured and analyzed using the cellSens Dimensions v.1.8. Software (Olympus, Tokyo, Japan). 

### 4.6. Immunofluorescence Assay

The morphological assessment of apoptotic cells in terms of nuclear localization and nuclear changes indicative of apoptosis (i.e., nuclear shrinkage/fragmentation) was performed using the 4,6′-Diamidino-2-Phenylindole (DAPI) staining. HaCaT and A375 cells were seeded in 6-well plates (1 × 10^6^ cells/well) and incubated until reaching 85–90% confluence. The old medium was then replaced with fresh medium, and the cells were stimulated with GNP and triterpene GNP conjugates (10, 25 and 50 μΜ) and incubated for another 24 h at 37 °C. After the stimulation period, the cells were washed 2–3 times with cold phosphate buffered saline PBS (1X) (Thermo Fisher Scientific, Boston, MA, USA) and fixed with paraformaldehyde 4% in PBS. The cells were then permeabilized with Triton X/PBS 2% for 30 min at room temperature, washed 2–3 times with cold PBS and blocked with 30% FCS in 0.01% Triton X for 1 h at room temperature. After another washing step with cold PBS, the cells were stained with DAPI (300 nM) and incubated at 4 °C in the dark overnight. The nuclear alterations were analyzed using a Zeiss Microscope AXIO Observer.D1 (Carl Zeiss Microscopy GmbH, Jena, Germany) equipped with the Snap-260—ZEN pro 2012. 

### 4.7. High-Resolution Respirometry 

Using high-resolution respirometry studies (Oxygraph-2k, Oroboros Instruments GmbH, Innsbruck, Austria), mitochondrial respiratory function was assessed at 37 °C. A modified substrate uncoupler-inhibitor titration (SUIT) protocol was followed in order to measure normal HaCaT and A375 melanoma cells’ mitochondrial respiration. As previously described by Petruș et al. [64], the measurement of respiratory rates was recorded for both separate and convergent Complex I and Complex II (CI + CII) electron input. Prior to these respirometry studies, the cells were cultured in T25 culture flasks until they reached 85–90% confluence and treated with triterpene GNP conjugates and GNP for 24 h. After the stimulation period, the cells were washed with PBS, trypsinized, counted and resuspended (1 × 10^6^/mL cells) in mitochondrial respiration medium (MIRO5: MgCl_2_ 3 mM, EGTA 0.5 mM, taurine 20 mM, KH_2_PO_4_ 10 mM, K-lactobionate 60 mM, D-sucrose 110 mM, HEPES 20 mM and BSA 1 g/L, pH 7.1). The concentration used was 25 μM, a concentration at which the tested compounds produced a significant decrease in A375 melanoma cell viability without decreasing that of HaCaT healthy cells.

The respirometric protocol started with the addition of cells in the chambers of the device containing free media (MIRO5), followed by a 15-min period of stabilization during which the first respiratory rate was recorded (routine respiration). After this time period, the modified SUIT protocol that was applied consisted of the addition of mitochondrial substrates and inhibitors and the subsequent measurement of mitochondrial respiratory rates, as follows:

(i) The addition of digitonine (35 μg/L × 10^6^ cells, a mild detergent that permeabilizes cell membrane in order to allow the passage of soluble molecules between external media and cytosol) and the CI substrates: glutamate (10 mM) and malate (5 mM) → measurement of basal respiration (State2_CI_).
(ii) ADP (5 mM) addition → measurement of active respiration dependent on CI (OXPHOS_CI_)
(iii) Succinate (10 mM), a CII substrate, is added to induce the maximal OXPHOS capacity of both CI and CII → measurement of OXPHOS_CI+CII_
(iv) Oligomycin (1 μg/mL), an inhibitor of complex V → measurement of LEAK respiration dependent on both CI and CII (State4_CI+II_)
(v) P-(trifluoromethoxy) phenylhydrazone carbonyl cyanide—FCCP (1 μM/step) successive titrations → to measure the maximal respiratory capacity of the electron transport system (ETS_CI+II_)
(vi) Rotenone (0.5 μM), a CI inhibitor → to measure the maximal respiratory capacity of the electron transport system dependent solely on CI (ETS_CI_)
(vii) Antimycin A (2.5 μM), a complex CIII inhibitor used to completely inhibit the electron transport system → measurement of residual oxygen consumption (ROX). All the obtained values were corrected after ROX.

### 4.8. Quantitative Real-Time PCR 

The Trizol reagent (Thermo Fisher Scientific, Inc., Waltham, MA, USA) and the Quick-RNA^TM^ purification kit were used to isolate total RNA (Zymo Research Europe, Freiburg im Breisgau, Germany). Total RNA was then transcribed using the Maxima^®^ First Strand cDNA Synthesis Kit (Thermo Fisher Scientific, Inc.). The Quant Studio 5 real-time PCR system (Thermo Fisher Scientific, Inc.) was used for quantitative real-time PCR analysis in the presence of Power SYBR-Green PCR Master Mix (Thermo Fisher Scientific, Inc.). Table 4 lists the primer pairs used (Thermo Fisher Scientific, Inc.). The comparative threshold cycle (2^−ΔΔCt^) method was used to calculate normalized relative expression data.

### 4.9. Apoptosis Assay

The apoptosis protein markers, BAX and Bcl-2, were analyzed after the treatment with 25 μΜ GNP and GNP-conjugated benzotriazolyl esters of betulinic, oleanolic and ursolic acids for 24 h. BAX (cat. no. ab199080) and Bcl-2 (cat. no. ab119506) concentrations in A375 cell lysates were determined using the ELISA kits acquired from Abcam, according to the manufacturers’ protocols. Optical densities were read using a microplate reader (xMark™ Microplate Spectrophotometer, Biorad, Hercules, CA, USA) at 450 nm. 

## 5. Conclusions

The GNP-conjugated benzotriazolyl esters of BA (OA-HOBt GNP), OA (OA-HOBt GNP), and UA (OA-HOBt GNP) were synthesized, characterized and biologically evaluated for their ability to inhibit the proliferation of human malignant melanoma. The citrate-capped gold nanoparticles (GNP) were synthesized by the reduction of chloroaurate using trisodium citrate, while GNP formation was validated by UV-VIS spectrometry. HaCaT healthy keratinocytes and A375 human melanoma cells were used to examine the conjugate’s cytotoxic effects. All three conjugates presented cytotoxic activity in a dose-dependent manner on A375 melanoma cells, whereas no cytotoxic effects were detected on normal HaCaT keratinocytes. Moreover, when compared to the parent compounds (BA-HOBt, OA-HOBt and UA-HOBt GNP), GNP-conjugates displayed a stronger cytotoxicity [17], thus implying that the metallic nanocarrier can facilitate an optimized cell delivery. Morphological changes of A375 melanoma cells consistent with apoptosis were detected through DAPI staining after the treatment with all three GNP-conjugates. RT-qPCR analysis showed a fold change increase in the expression of BAX and a decrease in the expression of Bcl-2, which occurred in the triterpene-GNP conjugate-treated A375 melanoma cells. These results were backed up by ELISA tests, which showed that all three samples induced a significant fold change in Bcl-2/BAX content in treated A375 cells. High-resolution respirometry studies revealed that all three GNP-conjugates act as selective inhibitors of mitochondrial function in A735 melanoma cells. All things considered, we can conclude that the GNP formulation of benzotriazole-triterpenic acid esters increases the antimelanoma effect of the unformulated compounds. Future work can establish if this increase in the antimelanoma effect caused by GNP functionalization is suitable for other types of triazole-bearing triterpene derivatives as well. This could pave the way for the derivatization and nanogold-based delivery of triterpenoid compounds.

## Figures and Tables

**Table 1:** Significantly assigned FTIR peaks of BA-HOBt GNP, OA-HOBt GNP and UA-HOBt GNP and their unformulated parent compounds.

Compound	FTIR Peak Assignment (cm^−1^)					
	O-H Stretch	C-H Stretch	Ester Group			sp^2^ Aromatic C-H Bend
			C=O Stretch	C-C-O Stretch	O-C-C Stretch	
BA-HOBt	3446	2945, 2872	1809	1240	1087	765, 742
BA-HOBt GNP	3445	2947, 2870	1809	1240	1087	766, 740
OA-HOBt	3441	2939, 2862	1811	1246	1087	783, 746
OA-HOBt GNP	3431	2939, 2862	1811	1246	1089	783, 752
UA-HOBt	3446	2928, 2872	1805	1242	1088	781, 742
UA-HOBt GNP	3433	2926, 2870	1805	1240	1085	781, 741

**Table 2:** Recorded hydrodynamic diameter, ζ potential and drug loading efficiency (DLE%) for GNP, BA-HOBt GNP, OA-HOBt GNP and UA-HOBt GNP.

Sample	Hydrodynamic Diameter (nm)		ζ Potential (mV) Mean ± SD	DLE% Mean ± SD
	Mean ± SD	PDI		
GNP	16 ± 0.8	0.07	−28.2 ± 1.1	-
BA-HOBt GNP	103 ± 7.3	0.20	−23.2 ± 0.7	34.2 ± 0.33
OA-HOBt GNP	99 ± 5.8	0.13	−24.0 ± 0.9	30.5 ± 0.87
UA-HOBt GNP	101 ± 6.7	0.16	−23.8 ± 0.5	33.8 ± 0.24

**Table 3:** Recorded FWHM and A_max_ at different time points for all three samples in PBS and DMEM.

Medium	PBS		DMEM	
Sample ID/Time Point	A_max_	FWHM	A_max_	FWHM
BA-HOBT GNP 1 h	530	95	530	95
BA-HOBT GNP 6 h	530	95	532	96
BA-HOBT GNP 12 h	530	95	532	97
BA-HOBT GNP 24 h	531	96	533	101
BA-HOBT GNP 48 h	530	96	535	105
BA-HOBT GNP 72 h	531	97	542	116
BA-HOBT GNP 96 h	531	99	559	129
OA-HOBT GNP 1 h	521	40	521	40
OA-HOBT GNP 6 h	521	40	521	42
OA-HOBT GNP 12 h	521	40	523	48
OA-HOBT GNP 24 h	523	40	524	52
OA-HOBT GNP 48 h	522	42	527	55
OA-HOBT GNP 72 h	522	42	538	83
OA-HOBT GNP 96 h	523	43	552	100
UA-HOBT GNP 1 h	528	61	528	61
UA-HOBT GNP 6 h	528	61	529	62
UA-HOBT GNP 12 h	528	61	529	63
UA-HOBT GNP 24 h	530	61	532	69
UA-HOBT GNP 48 h	530	62	535	81
UA-HOBT GNP 72 h	530	62	539	93
UA-HOBT GNP 96 h	530	62	545	114

**Table 4:** Primer pair used in rtPCR analysis.

Sequence ID	Forward	Reverse
18 S	5′ GTAACCCGTTGAACCCCATT 3′	5′CCA-TCC-AAT-CGG-TAGTAG-CG 3′
BAX	5′GGCCGGGTTGTCGCCCTTTT 3′	5′CCGCTCCCGGAGGAAGTCCA 3′
Bcl-2	5′CGGGAGATGTCGCCCCTGGT 3′	5′-GCATGCTGGGGCCGTACAGT-3′

## Data Availability

Data is contained within the article.

## Sample Availability

Samples of the compounds BA-HOBt GNP, OA-HOBt GNP and UA-HOBt GNP are available from the authors.
